# Continuous Glucose Monitoring as an Additional Tool in Early Cystic Fibrosis-Related Diabetes Monitoring and in Evaluation of Short-Term Sitagliptin Response

**DOI:** 10.3390/biomedicines11061754

**Published:** 2023-06-19

**Authors:** Fernando Sebastian-Valles, José Alfonso Arranz Martín, Rosa María Girón, Carolina Knott-Torcal, Miguel Antonio Sampedro-Nuñez, Jose Carlos Martin-Adan, Jessica Jiménez-Díaz, Mónica Marazuela

**Affiliations:** 1 Department of Endocrinology and Nutrition, Hospital Universitario de la Princesa, IIS-Princesa, Universidad Autónoma de Madrid (UAM), 28006 Madrid, Spain; 2 Department of Pneumology, Hospital Universitario la Princesa, IIS-Princesa, Universidad Autónoma de Madrid (UAM), 28006 Madrid, Spain

**Keywords:** CFRD, CGM, diabetes, gliptins, prediabetes, beta cell

## Abstract

Cystic fibrosis-related diabetes (CFRD) is a complication associated with a negative prognosis in patients with cystic fibrosis (CF). Although the oral glucose tolerance test (OGTT) is the widely recommended screening test for CFRD diagnosis, continuous glucose monitoring (CGM) is increasingly considered a useful and easy-to-perform test for diagnosis and follow-up in clinical practice. Regarding CFRD treatment, although insulin is the classic approved pharmacological option, incretins could also be a helpful alternative in early stages. CGM could be also a useful tool to measure the early response to this therapy. METHODS: We studied 25 CF patients with abnormal OGTT results and compared glucose and insulin levels during the OGTTs with CGM results as a tool for early CFRD diagnosis. In addition, we evaluated glycaemic control with CGM before and after treatment with sitagliptin. RESULTS: A correlation was found between lower plasma insulin levels during the OGTTs and higher average sensor glucose (*p* = 0.009) and hyperglycaemic excursions (*p* = 0.017). The CGM data on sitagliptin treatment (*n* = 25) showed an average glycaemic improvement from 124.2 to 117.2 mg/dL (*p* = 0.002) with a 5.6-point standard deviation of glucose decrease (*p* < 0.001). Hyperglycaemic excursions ≥200 mg/dL diminished 57.1% (*p* = 0.021). Both time in range and time above 180 mg/dL improved during treatment (*p* = 0.036 and *p* = 0.006, respectively). CONCLUSION: CGM is a useful tool that offers valuable information for both the diagnosis and the management of CFRD. Lower plasma insulin levels during OGTTs are associated with a poor ambulatory glucose profile in CGM. Sitagliptin could play an important role in the treatment of the early stages of CFRD.

## 1. Introduction

Cystic fibrosis (CF) is the most prevalent autosomal recessive genetic and life-limiting disease in the world population. Progressive respiratory impairment is the primary factor contributing to health complications. Pulmonary and nutritional outcomes serve as crucial indicators of overall health status in individuals with CF. The life expectancy of people with CF has notably changed in recent decades due to advances in respiratory, nutritional, and metabolic management. Their median age currently surpasses 50 years [1].

Cystic fibrosis-related diabetes (CFRD) is a frequent complication occurring in about 20% of adolescents and 40–50% of adults with CF [2]. It is associated with the worsening of respiratory function, as well as increases in morbidity and mortality [3,4]. Although there is evidence supporting the idea that pulmonary exacerbations can mediate the effect of CFRD on mortality, the complete extent of the effect remains to be determined [5]. Abnormal glucose tolerance identifies those patients at high risk for progression to early onset diabetes [6]. Moreover, respiratory and nutritional decline could begin in the prediabetes stage before CFRD detection [7,8], although this has not been confirmed in other recent studies [9].

The diagnosis of dysglycaemia in people with CF plays a key role in implementing appropriate therapeutic interventions capable of reducing the deleterious effect of this disease [10]. Thus, clinical guidelines recommend a yearly CFRD screening from the age of 10 years onwards. A standard 2 h oral glucose tolerance test (OGTT) is the only diagnostic test recommended for the detection of dysglycaemia in people with CF [4,11]. However, the OGTT is a time-consuming test with a high within-patient variability in CFRD diagnosis [12]. Alternative tests, such as glycosylated haemoglobin (HbA1c) tests, have not been validated to date as alternatives to the OGTT, as the data are still controversial [13,14]. Continuous glucose monitoring (CGM) is increasingly considered as an additional tool that could improve the early detection of pancreatic endocrine dysfunction [15] in people with CF. Nonetheless, to date it still remains a complementary test to the OGTT in CFRD diagnosis.

CFRD is a consequence of a dysfunctional beta-cell mass, which progressively worsens over the course of CF [16]. Accordingly, insulin therapy has proven to be superior to other pharmacological alternatives, because of its higher potential for glucose management as well as its anabolic effects. Although this is the rule when metabolic deterioration is evident, in the early stages of CFRD, when there is a minor metabolic deterioration and the nutritional status is not usually compromised, insulin therapy can represent a burden for people with CF, including by carrying the risk of hypoglycaemia, having higher costs, causing more stress, and requiring more education for its correct use.

As incretin axis dysfunction is thought to contribute to the development of glucose intolerance in CF [17], incretin therapy, specifically using dipeptidyl peptidase 4 inhibitors (DPP4i), which stimulate insulin secretion and suppress glucagon secretion in a glucose-dependent way, could be a good alternative in the early stages of CFRD. These agents, which are already approved for the treatment of type 2 diabetes mellitus in adults, could also be a very useful tool in the early stages of beta-cell pancreatic dysfunction with a very low risk of hypoglycaemia [18]. To date, there are very few studies assessing the role of DPP4i in CFRD; therefore, there is not enough evidence to recommend their use.

Here, we present an observational study with a prospective follow-up in adults with CF, who were followed at the Cystic Fibrosis Unit of our hospital. This study aims to assess the role of CGM as a complementary diagnostic tool in CF patients with the criterion of dysglycaemia. In addition, we used CGM as a method to assess early glycaemic response to sitagliptin as a proof of concept of the potential benefit of DPP4i in people with early CFRD or in prediabetic stages.

## 2. Materials and Methods

This prospective observational study enrolled adult patients (>18 years old) who were under follow-up in the Cystic Fibrosis Unit at Hospital Universitario de La Princesa in Madrid, Spain. Participants who exhibited abnormal results in OGTT were included. Written informed consent was obtained from all participants prior to their participation.

CF had been previously diagnosed through a genetic study of the CFTR mutation. In addition, all participants had been diagnosed with exocrine pancreatic insufficiency and were receiving treatment with proteolytic enzymes. Patients with previously diagnosed cystic fibrosis-related diabetes (CFRD) were excluded from the study. Other exclusion criteria were acute intercurrent processes, need for oral antibiotics in the 4 weeks prior to inclusion, oral or parenteral corticosteroid therapy 3 months preceding the baseline period, hepatic or renal disease, lung transplant, and pregnancy.

Anthropometric variables were collected, as well as genotype, CF duration, and daily dose of pancreatic enzymes. HbA1c was measured every time OGTT and/or CGM were performed. Percentage of predicted forced expiratory volume in 1 s (FEV1) and percentage of predicted forced vital capacity (FVC) were collected from the most recent office visit based on the date of the preceding CGM. Participants’ characteristics are displayed in Table 1.

### 2.1. Oral Glucose Tolerance Test

A 75 g OGTT was performed after overnight fast. Blood was collected through an indwelling catheter to analyse glucose and insulin levels every 30 min for 2 h (0, 30, 60, 90, and 120 min). We conducted an integrated analysis of OGTT plasma insulin levels at 0, 30, 60, 90, and 120 min using the trapezoidal rule to evaluate the area under the curve (AUC). Plasma insulin levels were measured in the UM Fairview Laboratory with radioimmunoassay using a double-antibody method (Immulite 2000, Siemens, Erlagen, Germany). Plasma glucose was measured using the glucose oxidase method (Vitros, Ortho-Clinical Diagnostics, Raritan, NJ, USA). Glycated haemoglobin was routinely determined using liquid chromatography (ADAMS A1c HA8180 V ARKRAY^®^).

Depending on the glucose levels during the OGTT, we established a classification according to current diagnostic criteria as follows: impaired fasting glycaemia (IFG): fasting glucose > 100 mg/dL and <126 mg/dL; indeterminate glycaemia (INDET): both fasting and 2 h glucose levels are within normal range but the glucose level is >200 mg/dL at another time point during the test; impaired glucose tolerance (IGT): 2 h glucose > 140 mg/dL and <200 mg/dL or CFRD if 2 h glucose > 200 mg/dL.

### 2.2. Continuous Subcutaneous Glucose Monitoring

Participants who displayed certain criteria indicative of glucose abnormalities were included in the study. A continuous subcutaneous glucose-monitoring device (either Dexcom G4 Platinum^®^ or Dexcom G6^®^) was employed as an additional diagnostic measure within a 3-month period subsequent to OGTT in each subject. The insertion and removal of the sensor were conducted by the nurse assigned to the Diabetes Unit within our department. Participants utilizing the Dexcom G4 Platinum^®^ were monitored for a duration of 14 days, while those using DEXCOM G6^®^ were monitored for 10 days. Throughout the duration, CGM participants were advised to maintain their usual dietary patterns. The CGM data were extracted and analysed using the corresponding platforms for each sensor, namely Dexcom Studio^®^ for Dexcom G4 and Clarity^®^ for Dexcom G6^®^.

Data interpretation was possible because of the lack of significant sensor data loss, since all participants displayed a data percentage greater than 80%. Participants who used Dexcom G4^®^ proved to have at least 2 calibrations daily, with additional capillary glucose determinations prior to their meals.

We defined dysglycaemia during CGM as the presence of at least two interstitial glucose values exceeding 200 mg/dl during the duration of sensor usage. We decided to use this value as the validated cut-off point, since it is the figure established for the diagnosis of diabetes in the biochemical criteria defined by the American Diabetes Association (ADA), although it is not validated in the AGP glucometric criteria.

These data were compared with the data from the OGTT, as well as with other clinical variables such as age, gender, type of CF-related dysglycaemia, glycosylated haemoglobin, homeostatic model assessment of insulin resistance (HOMA-IR), and daily dose (units/kg body weight) of oral pancreatic enzymes.

Results from the CGM were analysed in the hospital appointments by the senior endocrinologist responsible for the follow-up of patients with CF in our department, and were discussed together with the participants, and their carers if appropriate.

### 2.3. Sitagliptin Therapy

In the second half of the time of use of the sensor (after 7 days of use for Dexcom G4 and 5 days for Dexcom G6), a single daily dose of sitagliptin 100 mg was prescribed to be taken orally at midday.

CGM data were compared before initiating treatment with sitagliptin (OFF-STG) and during the course of treatment (ON-STG). Glucometric variables were collected as follows: average glycaemia; standard deviation (SD); time in range (TIR) 70–180 mg/dL; time above range (TAR); and time below range (TBR); as well as the number of glycaemic excursions with values above 200 mg/dL (Glucose ≥ 200) within each time period, corresponding to breakfast, lunch, dinner, and any snacks in between. This was adjusted according to the days of use of the sensor, and the differences between the two periods were analysed. All CGM results were adjusted for the varying sensor duration between the Dexcom G4 and Dexcom G6 sensors. The response to the sitagliptin treatment was determined by calculating the difference in average blood glucose levels and the number of hyperglycaemic peaks in CGM before and after sitagliptin administration.

### 2.4. Statistical Analysis

Data were analysed with the statistical software STATA 17.0 BE-Basic Edition (Lakeway Drive, College Station, TX, USA) and R, version 4.0.3. Quantitative variables were described with median and range, whereas qualitative variables were defined with frequency and percentage. Normality of the variables was assessed with the Shapiro–Wilk test. Subsequently, differences between groups were tested with Mann–Whitney U test and quantile regression of medians for non-normal variables and Student’s *t*-test for normal variables. For qualitative variables, the chi-squared test was used. To assess the correlation between variables, the Pearson’s r and Spearman’s rho tests were used for normally and non-normally distributed variables, respectively. *p* values < 0.05 were considered significant.

### 2.5. Ethics

The study protocol was approved by the local Ethics Committee (number 4794—05/22). The research was conducted according to the Declaration of Helsinki.

## 3. Results

We evaluated the results of the OGTTs and CGM in a cohort of 25 participants with CF. The median age was 26 years (range 18–56), and 68% were men (*n* = 17). Seventeen participants (68%) used Dexcom G6^®^ and eight (32%) used Dexcom G4 Platinum^®^. All participants received enzymes for exocrine pancreatic insufficiency. Seventeen patients (68%) met the OGTT diagnostic criteria for CFRD; two of them presented basal glucose levels >126 mg/dL in previous blood test results. The other eight subjects were in a prediabetic stage: three (12%) met the criteria for IGT, and five (20%) were diagnosed with INDET. The average global HbA1c was 5.9%, corresponding to 5.9% and 5.8% in patients with diabetes and prediabetes, respectively. Other descriptive variables are displayed in Table 1.

### 3.1. Basal CGM Results

The basal average blood glucose in CGM was 124.2 ± 33.1 mg/dL. The average glucose was 122.2 ± 15.5 mg/dL in patients with prediabetes criteria and 125.2 ± 15 mg/dL in those with diabetes criteria (*p* = 0.656).

The number of hyperglycaemic peaks was adjusted based on the number of days of sensor usage. The number of glucose excursions above 200 mg/dL observed was 1.36/day on average, 1.44/day and 1.19/day in patients with diabetes and prediabetes, respectively (*p* = 0.539) (1.3 excursions/day in the IGT subgroup and 1.1/day in the INDET subgroup). The percentages of TIR, TAR, and TBR could only be assessed in participants who wore the Dexcom G6, since the Dexcom G4 platinum does not include these measurements. The TIR of our cohort was 88.7%, 91.1% in the prediabetes group and 87.8% in the diabetes group. In our cohort, 10.1% of patients had a TAR > 180 mg/dL and 1.3% TAR > 250 mg/dL. Data downloaded from the sensor showed a TBR < 70 mg/dL in 1.22% of patients, which was not associated with hypoglycaemia signs or symptoms in any patient. A TBR < 54 mg/dL was not observed. None of the differences between the different subtypes were statistically significant.

**Figure 1:**
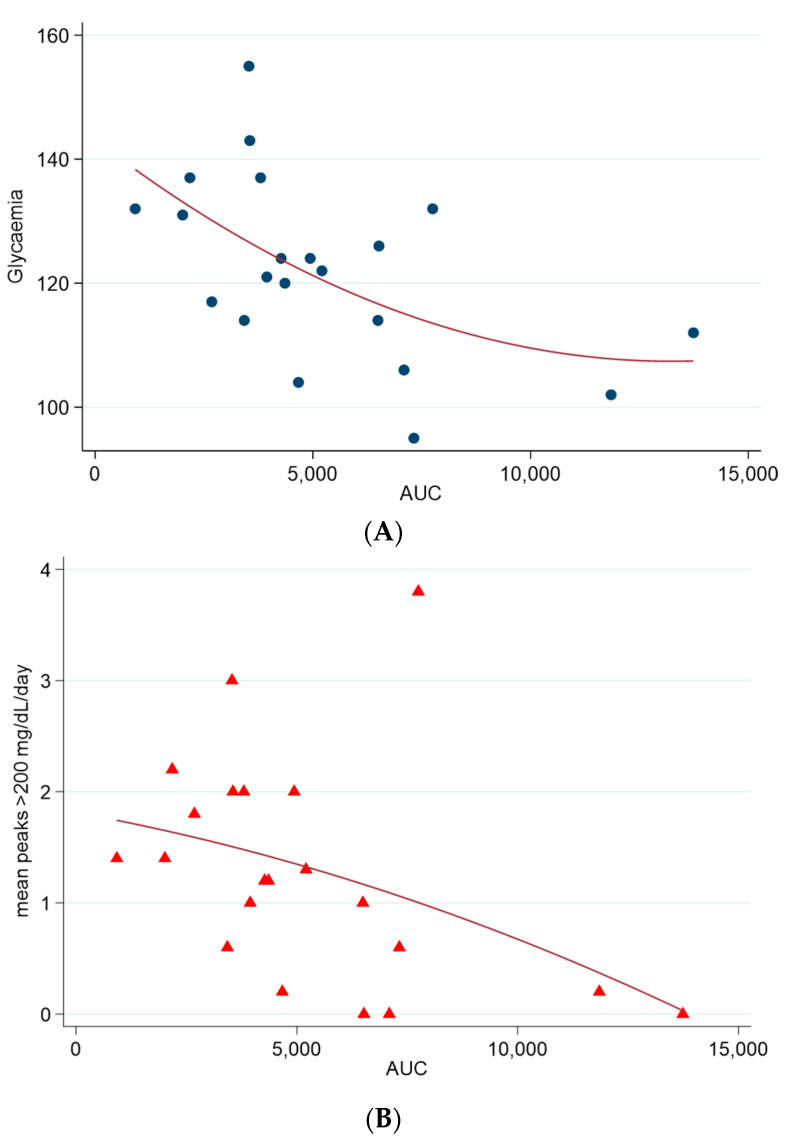
Correlation graph between the AUC of insulinemia and the following: (**A**) mean glycaemia in CGM sensor (Spearman’s rho = −0.567; *p* = 0.009) and (**B**) mean hyperglycaemic peaks > 200 mg/dL in CGM sensor (Spearman’s rho = −0.515; *p* = 0.017).

Additionally, we found a correlation between insulin plasma concentrations during the OGTT, the average glucose, and the hyperglycaemic excursions obtained from the sensor records during the basal period of CGM. We conducted an integrated analysis of OGTT plasma insulin levels at 0, 30, 60, 90, and 120 min using the trapezoidal rule to evaluate AUC. A moderate association was observed between plasma insulin levels and average sensor glucose (Spearman’s rho = −0.567; *p* = 0.009), as well as an association between plasma insulin levels and glucose ≥ 200 mg/dL values (Spearman’s rho = −0.515; *p* = 0.017) (Figure 1). Lower insulin levels correlated with a worse average glycaemic profile and postprandial peaks during CGM. These findings are consistent with impaired secretion as a pathophysiological substrate of CFRD.

No associations were found between gender, age, genotype, the results of the OGTT, and CGM.

In the present study, no statistically significant differences were observed in the quantity of pancreatic enzyme treatment administered. Similarly, no differences were found for HbA1c or HOMA-IR.

### 3.2. Results of CGM during Sitagliptin Therapy

CGM data before and after sitagliptin therapy are shown in Table 2. The data ON-STG revealed an average glucose level of 117.2 mg/dL (−7 mg/dL compared with the basal OFF-STG value, 124.2 mg/dL) (*p* = 0.002; 95% CI [2.89–11.11]). The number of hyperglycaemic excursions ≥ 200 mg/dL was 0.6 peaks/day, a 57.1% decrease vs. that of OFF-STG, which was 1.4 peaks/day (*p* = 0.021; 95% CI [0.1–1.3]). The SD of glucose levels showed a significant average decrease of 5.6 points (*p* < 0.001; 95% CI [3.15–8.05]). Glucometrics results showed a slight significant 3.46% improvement in TIR (*p* = 0.036; 95% CI [6.67–0.26]) from 88.7% to 92.2%. TAR > 180 mg/dL improved by 4.9% (*p* = 0.006; 95% CI [1.38–6.80]) from 10.1% to 6.0%. TAR > 250 mg/dL presented a mild decrease (*p* = 0.011; 95% CI [0.07–2.15]) from 1.3% to 0.5%. TAR < 70% showed a nonsignificant increase from 1.2% to 1.7% (*p* > 0.05).

In addition, an inverse association was observed between AUC and the efficacy of sitagliptin in average blood glucose levels (rho = −0.459, *p* = 0.0362), as well as in the number of hyperglycaemic peaks exceeding 200 mg/dL (rho = −0.559, *p* = 0.008).

**Figure 2:**
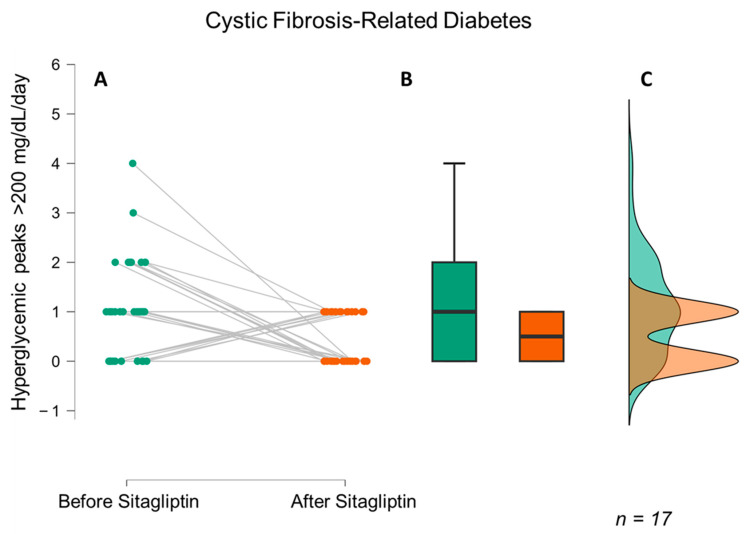
Median hyperglycaemic peaks > 200 mg/dL/day before and after treatment with sitagliptin in CFRD (*p* < 0.001). In green, we present the number of daily hyperglycaemic peaks > 200 mg/dL prior to sitagliptin administration, and in red, we display the number of daily hyperglycaemic peaks > 200 mg/dL during treatment with sitagliptin. (**A**,**B**) depict the decrease in the number of daily hyperglycaemic peaks > 200 mg/dL in a scatter plot and box plot, respectively, indicating a significant reduction in the number of daily peaks. (**C**) displays a raincloud plot, demonstrating a decrease in the distribution of daily peaks between 0 and 1 during treatment with sitagliptin.

**Figure 3:**
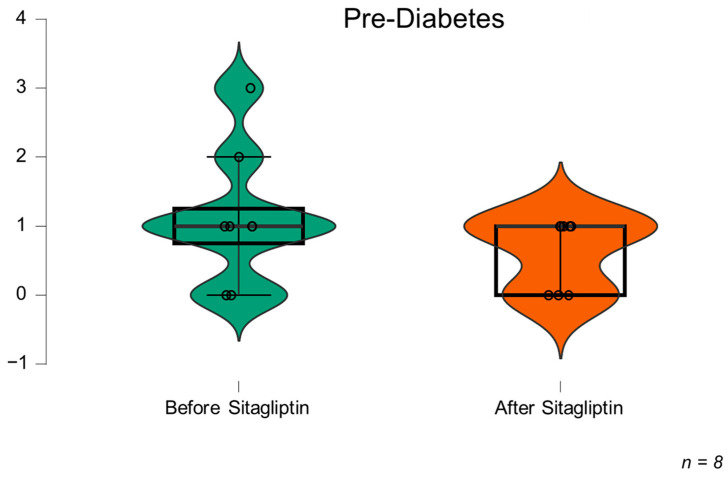
Median hyperglycaemic peaks > 200 mg/dL/day before and after treatment with sitagliptin in prediabetes. Prediabetes is defined by an oral glucose tolerance curve with impaired glucose tolerance or indeterminate glucose alteration (*p* = 0.08).

Taken all together, these findings show that sitagliptin decreases glycaemic peaks in CFRD (Figure 2) and shows a trend in pre-DM (Figure 3).

## 4. Discussion

This study aimed to analyse the role of CGM in the diagnosis of early stage CFRD and to compare the degree of agreement and possible complementarity of this tool with the standard test (OGTT). In addition, we also evaluated the possible use of CGM as an assessment tool to evaluate short-term glycaemic response to CFRD treatment, which in this study specifically focused on the response to treatment with the DPP4i sitagliptin.

The early detection of endocrine pancreatic insufficiency is a relevant matter because it can minimize the impact of CFRD on weight loss, lung function impairment, and mortality rates. CF clinical practice guidelines recommend screening all individuals annually with the OGTT, starting at the age of 10. However, this recommendation frequently has a suboptimal implementation in clinical practice. Data from the CF Foundation Registry point out that less than 40% of adults with CF have an OGTT performed on an annual basis [1]. Hence, in recent years a new interest in studying other diagnostic tools such as CGM has emerged, mainly as an alternative to OGTTs. CGM has mainly been validated in CF in children and adolescents [19,20], and several studies have shown that some specific measurements such as early glucose abnormalities detected by CGM are associated with poor health outcomes [21,22,23] in this population. Despite the limited data on CGM in adults with CF, it seems that early glycaemic alterations that can be detected with sensors could be linked to poorer clinical outcomes [24,25].

The data from our study show similar results in CFRD diagnosis between CGM and the OGTT if excursions ≥ 200 mg/dL during CGM are considered as a criterion of endocrine pancreatic insufficiency. All participants included in the study with abnormal OGTT results also presented glucose ≥ 200 mg/dL in CGM, except one (who had IGT criteria in the OGTT). Interestingly, in our study we identified a negative correlation between the plasma insulin response during the OGTT, evaluated using an integrated analysis such as AUC, and various parameters measured during the CGM basal period. Furthermore, CGM could provide some advantages over the OGTT, as CGM reflects the glycaemic response as a continuous variable in the daily routine, which is based on the typical nutrient intake of the patient through the day and not on a single glucose intake. Additionally, this could also be used as an educational tool with therapeutic purposes.

There is some evidence suggesting that early treatment of cystic fibrosis-related dysglycaemia could be beneficial in preventing or delaying the progression to cystic fibrosis-related diabetes [26,27]. However, the evidence is limited, and there is no consensus on the appropriate timing of treatment initiation or the most effective approach. Although the efficacy of insulin therapy cannot be doubted when the nutritional status is compromised or when metabolic deterioration is evident, this evidence is less consistent in prediabetes stages [27,28,29]. CF patients are usually reluctant to undergo insulin therapy in relation to its route of administration, the risk of hypoglycaemia, and the necessity of monitoring, among other causes which frequently lead to poor treatment compliance. Therefore, oral treatment modalities remain an interesting option because of their lower treatment burden and their lower risk of hypoglycaemia when compared with insulin. In these early stages, some CF units use oral medications to help in the management of diabetes, but the clinical practice guidelines of the CF Foundation do not support this approach. Currently, the vast majority (71%) of individuals diagnosed with CFRD are treated with insulin therapy according to the CF Foundation Registry, whereas only 4% are treated with oral agents [4]. There are already studies comparing the effectiveness of insulin and oral agents, particularly repaglinide, in patients with CFRD in relation to blood sugar levels, lung function, and weight management [30,31].

Sitagliptin is an antidiabetic drug that inhibits dipeptidyl peptidase-4 (DPP-4). DPP-4 is an enzyme found on the surface of various cells, which can prevent the degradation of incretins, including glucagon-like peptide-1 (GLP-1) and gastric inhibitory peptide (GIP), thereby producing an antihyperglycaemic effect [32]. The potency of different DPP-4 inhibitors such as vildagliptin, saxagliptin, and sitagliptin has been studied in randomized clinical trials in type 2 diabetes patients and a significant improvement in glycaemic control has been observed without differences between the different drugs, suggesting that their antihyperglycaemic potency constitutes a pharmacological class effect [33]. In addition, sitagliptin has beneficial effects on diseases derived from complications of diabetes mellitus, such as ischaemic heart disease [34], atherosclerosis, and hypertension [35]. Sitagliptin also appears to be useful in the initial treatment of microangiopathic complications of diabetes, such as diabetic retinopathy [36,37,38] and diabetic nephropathy [39], in experimental models. In addition, sitagliptin appears to have pleiotropic effects, including effects on COVID-19 infection. In this regard, there is clinical evidence from observational studies showing that treatment with sitagliptin at the time of hospitalization of patients with COVID-19 pneumonia is associated with reduced mortality and improved clinical outcomes compared with standard treatment [40]. We chose this pharmacological group because of its safety profile [41], mechanism of action, simplicity of use, and tolerability. In people with CF and dysglycaemia, sitagliptin intervention seems to enhance meal-related incretin responses with improved early insulin secretion and glucagon suppression without affecting postprandial glycaemia or glycated haemoglobin levels [42,43,44].

The ability to increase insulin secretion through the incretin effect of DPP-4 inhibitors has been described not only in type 2 diabetes [19] but also in CFRD [44]. In this regard, we found a negative association between plasma insulin response during the OGTT, evaluated using AUC, and the effectiveness of sitagliptin in reducing mean blood glucose levels and glycaemic excursions in CFRD. This finding suggests that the incretin effect is more effective in individuals with greater pancreatic secretion impairment, and does not produce significant glycaemic changes in individuals with better secretion and milder glycaemic alteration.

Daily CGM records could be an interesting tool to analyse a possible pharmacological response to oral therapy in CF patients with dysglycaemia. The identification of this response could contribute to earlier treatment with oral therapy, leading to the potential advantages of earlier better glycaemic control. This is the first study that examines the effects of sitagliptin using CGM in an adult population with CF. Our results show a significant reduction in glucose values, as well as significant differences in the parameters recommended by the current consensus on glucometric variables, without increasing the time of hypoglycaemia [26]. This improvement in CGM data during sitagliptin therapy was observed independently of the dysglycaemia criteria based on OGTT results. Although further studies are necessary, CGM data could be a valuable tool for detecting inadequate beta-pancreatic secretion and could serve to identify individuals who may benefit from treatment with DPP-4 inhibitors in the early stages of CFRD.

However, our study has some limitations. Firstly, the limited number of subjects precludes establishing firm conclusions. Secondly, our study lacks a control group without pharmacological intervention to compare these results. Thirdly, DEXCOM G4 and DEXCOM G6 Mean Absolute Relative Differences (MARDs) are not comparable. Lastly, the registered time under treatment with sitagliptin was too short to properly validate the glucometric results.

## 5. Conclusions

CGM data can provide valuable information for the diagnosis and personalized management of CFRD. Lower plasma insulin levels during OGTTs are associated with poorer ambulatory glucose profiles in CGM and poorer responses to sitagliptin. Sitagliptin therapy may play an important role in the treatment of the early stages of dysglycaemia and CFRD with good tolerance and without the risk of hypoglycaemia. This treatment can produce a rapid improvement in glucometric parameters that can be monitored using CGM. However, it remains to be determined whether DPP4 inhibitors have potential long-term effects on metabolic and clinical outcomes compared with insulin therapy. Ultimately, larger multicentre studies will help to establish more consistent evidence in this field.

## Figures and Tables

**Table 1:** Clinical characteristics of the participants.

Characteristic	
Age	26 (18)
Men/Women	17/8 (68%/32%)
HbA1c %(mmol/mol)	5.9% (0.4)
HOMA-IR	2.04 (0.48–5.33)
Homozygosis F508 mutation	11 (44%)
Heterozygosis F508 mutation	10 (40%)
Other mutations	4 (16%)
FEV1%	72 (31)
FVC%	87 (19)
FEV1/FVC	70 (10)
Impaired fasting glucose	0
Impaired glucose tolerance	3 (12%)
Indeterminate glycaemia	5 (20%)
Diabetes mellitus	17 (68%)
Pancreatic enzymes u/kg/day	3584 (2975)
Fasting glucose, mg/dL	101 (20)
1 h OGTT glucose, mg/dL	258 (83)
2 h OGTT glucose, mg/dL	214 (102)

Categorical data are presented as absolute numbers and percentages. Continuous data are presented as median and interquartile ranges 25–75. Abbreviations: HOMA-IR: homeostasis model assessment-estimated insulin resistance. FEV1: Forced expiratory volume in the first second. FVC: Forced vital capacity. OGTT: oral glucose tolerance test.

**Table 2:** CGM results before and during sitagliptin treatment.

Result	OFF-STG	ON-STG	*p*-Value
Glycaemia average (mg/dL)	124.2 ± 14.9	117.2 ± 13.8	*p* = 0.002 95% CI [2.9–11.1]
SD	33.1 ± 9.1	27.5 ± 8.3	*p* < 0.001 95% CI [3.2–8.1]
Number of hyperglycaemic peaks > 200 mg/dL/day	1.4 (0.6–2)	0.6 (0.3–1)	*p* = 0.021 95% CI [0.1–1.3]
TIR (70–180 mg/dL)%	88.7 ± 6.1	92.2 ± 5.2	*p* = 0.036 95% CI [6.7–0.3]
>180 mg/dL %	10.1 ± 6.8	6.0 ± 4.6	*p* = 0.006 95% CI [1.4–6.8]
>250 mg/dL %	1.3 ± 2.2	0.5 ± 1.1	*p* = 0.011 95% CI [0.1–2.2]
<70 mg/dL %	1.2 ± 2.3	1.7 ± 3.4	*p* > 0.05

Abbreviations: CI: confidence interval. OFF-STG: basal CGM data before sitagliptin administration. ON-STG: CGM data during sitagliptin administration. SD: standard deviation. TIR: time in range.

## Data Availability

Some or all datasets generated during and/or analysed during the current study are not publicly available, but are available from the corresponding author on reasonable request.
